# p53 is active in murine stem cells and alters the transcriptome in a manner that is reminiscent of mutant p53

**DOI:** 10.1038/cddis.2015.33

**Published:** 2015-02-26

**Authors:** H Yan, V Solozobova, P Zhang, O Armant, B Kuehl, G Brenner-Weiss, C Blattner

**Affiliations:** 1 Karlsruhe Institute of Technology, Institute of Toxicology and Genetics, Karlsruhe, Germany; 2 University of Heidelberg, Heidelberg, Germany; 3 Karlsruhe Institute of Technology, Institute of Functional Interfaces, Karlsruhe, Germany

## Abstract

Since it was found that p53 is highly expressed in murine embryonic stem cells, it remained a mystery whether p53 is active in this cell type. We show that a significant part of p53 is localised in the nucleus of murine embryonic stem cells and that the majority of this nuclear p53 is bound to DNA. According to its nuclear localisation, we show that p53 alters the transcriptional program of stem cells. Nevertheless, the anti-proliferative activity of p53 is compromised in stem cells, and this control is due, at least in part, to the high amount of MdmX that is present in embryonic stem cells and bound to p53. Instead of the anti-proliferative activity that p53 has in differentiated cells, p53 controls transcription of pro-proliferative genes in embryonic stem cells including *c-myc* and *c-jun*. The impeded anti-proliferative activity of p53 and the induction of certain proto-oncogenes by p53 in murine embryonic stem cells can explain why stem cells proliferate efficiently despite having high levels of p53.

The tumour suppressor protein p53 is a transcription factor that induces proliferation arrest and cell death via nuclear and cytoplasmic activities.^1, 2^ Under conditions with an increased risk of mutagenesis, p53 levels rise and the tumour suppressor protein becomes post-translationally modified, resulting in its activation.^3^ Cells lacking p53 usually proliferate faster than their wild-type counterpart even in the absence of stress^4^ indicating that p53 is active to some extent even under normal growth conditions.

p53 is frequently mutated in human tumours.^5^ Intriguingly, tumour-derived mutant *p53* usually not only lost wild-type activities but frequently enhances cell proliferation and invasiveness,^6, 7^ which is reflected by an altered p53-dependent transcriptional program.^6, 7^

Murine embryonic stem cells (mESCs) are pluripotent cells that usually proliferate fast and have a high amount of p53.^8^ This raises the questions how mESCs can proliferate so fast and why mESCs have so much p53.

We show that the anti-proliferative activity of p53 is compromised in mESCs. In mESCs, p53 is associated with MdmX, which controls its anti-proliferative activity. A fraction of p53 with a neutral pI is exclusively present in mESCs. In mESCs, p53 directs a transcriptional program that is highly reminiscent to that of tumour-derived mutant p53.

## Results

### p53 is primarily nuclear in mESCs

**Figure 1:**
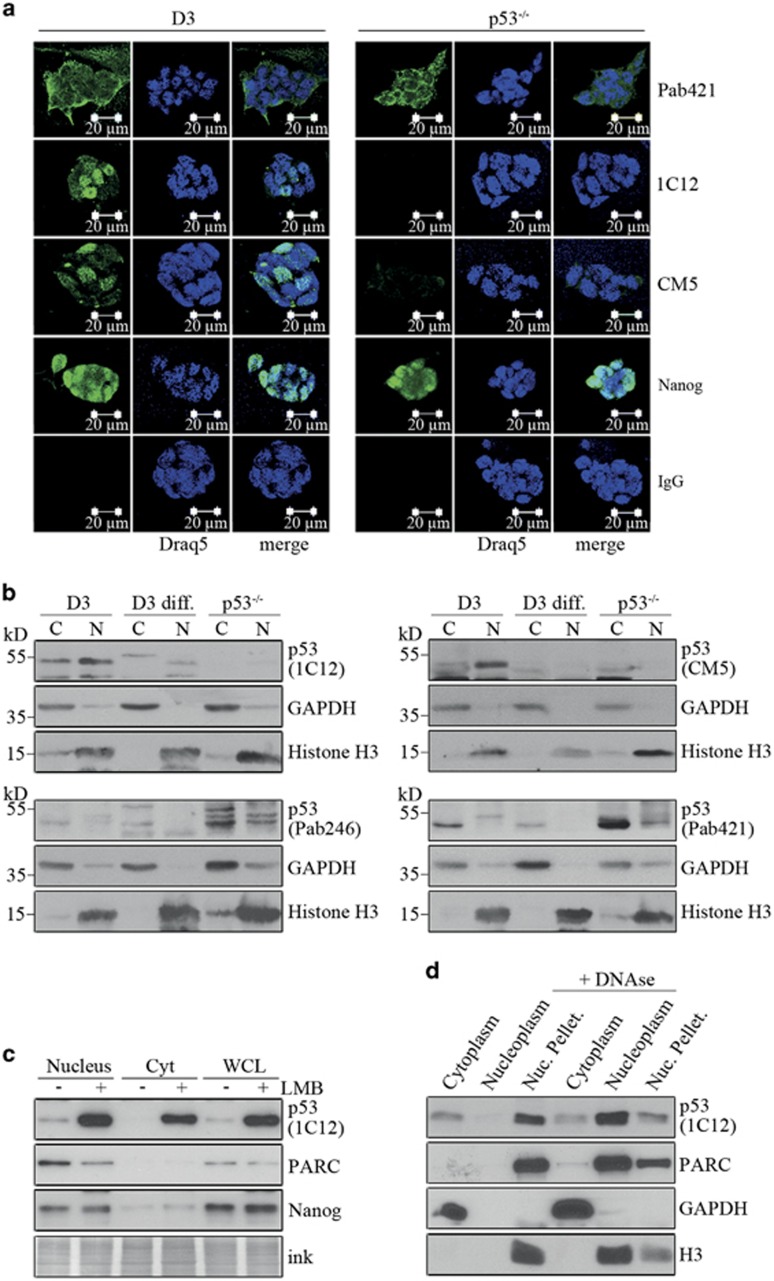
The majority of p53 is localised in the nucleus in murine embryonic stem cells. (**a**) D3 embryonic stem cells and their p53-deficient derivative (p53^−/−^) were grown on feeder cells on cover slips. Cells were fixed, stained with the indicated antibodies (shown in green) and counterstained with the nuclear marker Draq5 (shown in blue). Staining without primary antibodies (IgG) was performed for control. Images were analysed on a Leica LSM microscope. (**b**) D3 cells, their p53-deficient derivative (p53^−/−^) and D3 cells that had been differentiated with retinoic acid (D3 diff.) were fractionated into cytoplasmic and nuclear lysate. Lysates corresponding to an equal amount of cells were loaded onto SDS-PAGE gels and blotted. Four identical membranes were prepared and each of them was incubated with a different anti-p53 antibody (1C12, CM5, Pab246, Pab421). Abundance of GAPDH and Histone H3 was used to monitor successful fractionation. (**c**) Cells were serum-starved for 24 h prior to treatment with 2 *μ*M leptomycin B (LMB) for 16 h. An aliquot of the cells was lysed and used to monitor abundance of p53 in the whole-cell extract (WCL). The remaining cells were fractionated into cytoplasmic and nuclear lysate. Forty micrograms of the different fractions were loaded onto an SDS-PAGE gel and blotted. The membranes were hybridised with the anti-p53 antibody 1C12 and with anti-Nanog and anti-PARC antibodies as nuclear markers. Staining with ink was performed to monitor equal loading of the gel. (**d**) D3 cells were fractionated into a cytoplasmic and a nuclear part. One part of these fractions was incubated with 200 units/ml DNAseI or mock-treated for 1 h. After incubation, the nuclear fraction was cleared by centrifugation. Abundance of p53 in the different fractions was monitored by western blotting. PARC and Histone H3 were used as nuclear markers and GAPDH as a cytoplasmic marker

p53 is an anti-proliferative protein and highly abundant in mESCs (Supplementary Figure S1A),^8^ a cell type that proliferates faster than most differentiated cell lines (Supplementary Figure S1B). This observation raised the question how mESCs can proliferate so efficiently despite having high amounts of p53. One argument that was used in the past is that p53 would be cytoplasmic in stem cells. We monitored p53 localisation by immunofluorescence staining with four different anti-p53 antibodies. For control, we employed p53^−/−^ mESCs that were derived by gene targeting and are thus genetically identical with our p53-positive D3 stem cells. In agreement with previous studies,^9, 10^ we observed staining in the cytoplasm with the anti-p53 antibodies Pab421 and Pab246. Surprisingly, these antibodies gave signals of similar intensity also in the cytoplasm of p53^−/−^ mESCs (Figure 1a,Supplementary Figure S2A). Only when we used the anti-p53 antibody 1C12, we did not see any staining in p53^−/−^ cells. When we applied the anti-p53 antibody CM5, we only occasionally got a very weak staining. Importantly, with the 1C12 and CM5 antibodies, the majority of the staining was in the nucleus although not all p53-positive cells were stained with the same intensity (Figure 1a, Supplementary Figure S2A). To confirm these results, we fractionated mESCs into cytoplasmic and nuclear lysate. In addition, we included mESCs that had been differentiated with retinoic acid. To control for the efficiency of cell fractionation, we monitored abundance of the nuclear protein Histone H3 and the cytoplasmic protein GAPDH. We prepared four identical membranes onto which we had loaded an equal number of cells of the different cell types. In agreement with the immunofluorescence analysis, the antibodies Pab246 and Pab421 showed a strong signal in the cytoplasm of p53-positive stem cells (Figure 1b). This signal, however, was also present in p53-negative cells (Figure 1a). Only the antibodies CM5 and 1C12 recognised a protein of a molecular weight of about 53 kD that was absent in p53^−/−^ mESCs. The majority of this protein was in the nucleus, confirming the result from the immunofluorescence staining. Nevertheless, there was also some p53 in the cytoplasm (Figure 1b), showing that p53 is present both in the cytoplasm and nucleus of mESCs. In differentiated cells, we only detected p53 in the nucleus; most likely because of the much lower amount of p53 in this cell type and the low sensitivity of the assay (Figure 1b).

To further support the finding that p53 is nuclear in mESCs, we treated cells with leptomycin B, a drug that inhibits CRM1-dependent protein export and leads to nuclear accumulation of p53.^11, 12^ If p53 would be purely cytoplasmic in mESCs, this drug should prevent nuclear accumulation of p53. However, treatment of mESCs with leptomycin B resulted in a strong accumulation of p53 both in the nucleus and cytoplasm of mESCs (Figure 1c,Supplementary Figure S2B).

Transcription factors usually have a high affinity for DNA. Thus, if p53 would be cytoplasmic, it should not associate with DNA. To test this rationale, we fractionated cells into cytoplasm and nuclei, lysed the nuclei, divided the lysate into two parts and added DNAseI to one part. We then incubated both parts for 1 h on ice. We pelleted the insoluble components of the nuclei and monitored abundance of p53 in all fractions by western blotting. Again, most p53 was nuclear. Without DNAse treatment, p53 was exclusively in the insoluble nuclear material (Figure 1d), whereas treatment with DNAseI released p53 into the soluble nuclear part (Figure 1d), indicating that most p53 in mESCs is bound to DNA.

### The activity of p53 is compromised in embryonic stem cells

**Figure 2:**
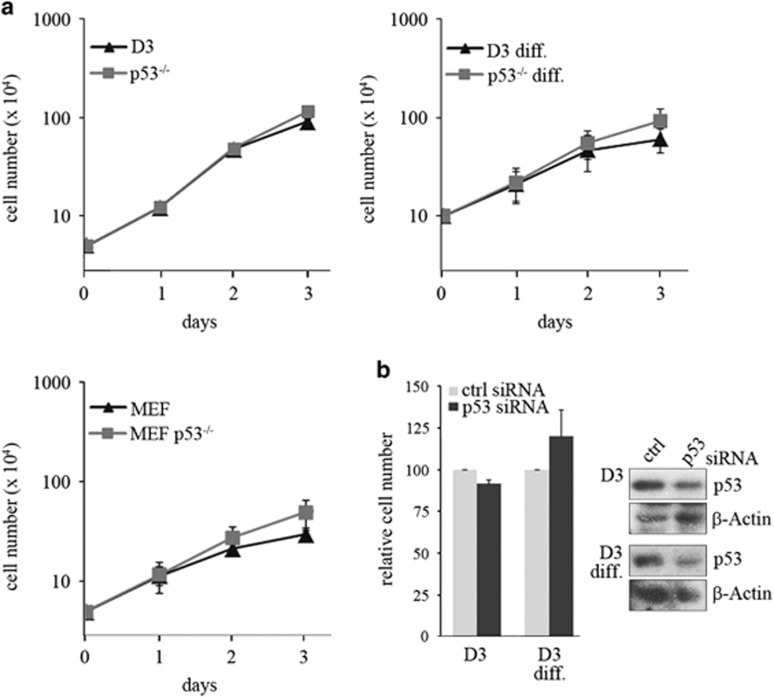
The anti-proliferative activity of p53 is compromised in stem cells. (**a**) D3 cells, their p53-deficient derivative (p53^−/−^), mouse embryonic fibroblasts (MEF) and their p53-deficient counterpart (MEF p53^−/−^) were plated at a density of 5 × 10^4^ cells/well in a 6-well plate. For differentiated D3 cells (D3 diff.), D3 cells and p53-negative cells were plated at a density of 10^5^ cells/well in 6-well plates. At the time of plating, retinoic acid was added to differentiate the cells. All cells were counted each day. The graph shows mean values and standard deviations of three independent experiments. (**b**) D3 cells and D3 cells that had been differentiated with retinoic acid for seven days (D3 diff.) were transfected with a siRNA targeted against p53 or with a control siRNA in triplicates. Seventy-two hours after transfection, relative numbers of living cells were determined by MTT assay from two of the duplicates. The graph shows mean values and error bars of two (D3 diff.) or three (D3) independent experiments. The third triplicate of the cells was used to monitor abundance of p53 by western blotting. Hybridisation with *β*-Actin was performed for loading control

Another explanation for the rapid proliferation of stem cells would be that p53 is inactive in mESCs. As p53 reduces proliferation of differentiated cells even under normal culture conditions,^4^ we monitored proliferation of p53-positive and p53-negative mESCs. For control, we employed p53-positive and p53-negative murine embryonic fibroblasts (MEFs) and differentiated mESCs. In consistency with Li and co-workers,^4^ we observed that p53-negative differentiated cells proliferated considerably faster than their wild-type counterpart. A difference in cell number was already discernible 2 days after plating, and this difference increased further in the following days. In contrast, we did not observe a difference in the number of p53^−/−^ and p53^+/+^ ESCs up to 2 days after plating (Figure 2a). This result was largely confirmed by MTT assays. Here, we observed a slightly higher signal with p53^−/−^ ESCs in comparison with p53^+/+^ mESCS, but the difference was much smaller than the difference between p53^+/+^ and p53^−/−^ fibroblasts (Supplementary Figure S3). Moreover, only differentiated cells proliferated faster after downregulation of p53 but not stem cells (Figure 2b). Likewise, nutlin, a compound that induces p53 abundance and activity^13^ only reduced proliferation of p53-positive differentiated cells, but not of stem cells (Supplementary Figure S3B). Altogether, these results show that the anti-proliferative activity of p53 is compromised in mESCs.

### p53 with a neutral pI is exclusively present in mESCs

**Figure 3:**
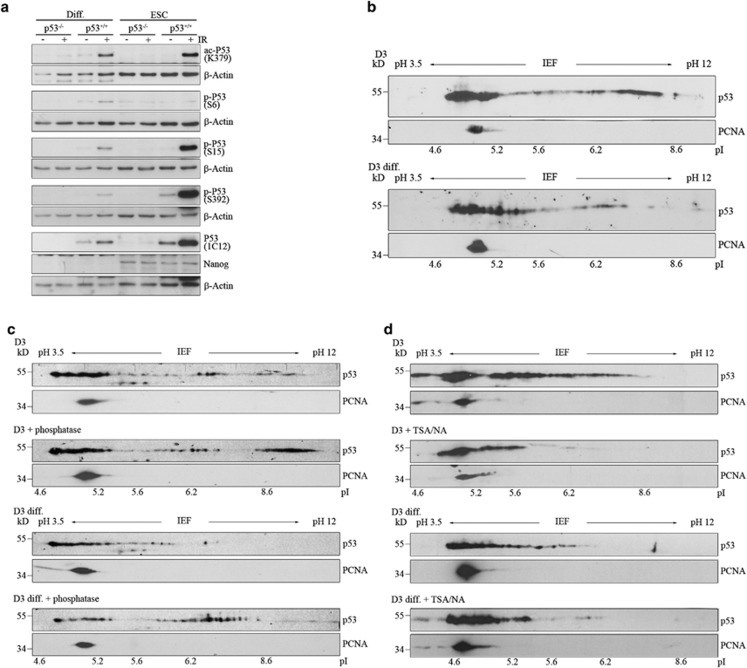
A fraction of p53 with a neutral pI exists exclusively in stem cells. (**a**) D3 cells (ESC), D3 cells that had been differentiated with retinoic acid for 7 days (Diff.) and their p53-negative counterparts were irradiated with 7 Gray or left un-irradiated. Two hours after irradiation, cells were lysed. The lysates were separated on five SDS-PAGE gels and blotted. Each membrane was hybridised with a different anti-p53 antibody, either directed against p53 that has been phosphorylated or acetylated at the indicated sites or against pan-p53. Hybridisation with Nanog was performed to control for stemness and with *β*-Actin to control for equal and comparable loading. Western blots were developed by ECL. (**b**) D3 cells (ESC) and D3 cells that had been differentiated with retinoic acid for 7 days (D3 Diff.) were lysed, separated by two-dimensional gel electrophoresis and blotted. Abundance of p53 and of PCNA, for internal control, was monitored by western blotting. (**c**) D3 cells (ESC) and D3 cells that had been differentiated with retinoic acid for 7 days (D3 Diff.) were treated with lambda phosphatase or mock-treated for control. The proteins were TCA-precipitated, separated by two-dimensional gel electrophoresis and blotted. Abundance of p53 and of PCNA, for internal control, was monitored by western blotting. (**d**) D3 cells (ESC) and D3 cells that had been differentiated with retinoic acid for 7 days (D3 Diff.) were treated with 1 *μ*M trichostatin A (TSA) and 5 mM nicotinamide (NA) for 6 h. Cells were lysed, separated by two-dimensional gel electrophoresis and blotted. Abundance of p53 and of PCNA, for internal control, was monitored by western blotting

p53 activity is largely controlled by post-translational modifications.^3^ We therefore wondered whether p53 might be differently modified in mESCs and differentiated cells. To investigate this, we determined the amount of modified p53 using antibodies directed against acetylated lysine 379 (K379), phosphorylated serine 6 (S6), phosphorylated serine 15 (S15) or phosphorylated serine 392 (S392). For control, we included p53-negative cells and cells that had been irradiated with ionising radiation. After DNA damage, p53 was acetylated at K379 and phosphorylated at S15 and S392, confirming the reactivity of the antibodies. None of the antibodies showed any signal in p53^−/−^ cells, showing their specificity (Figure 3a). Apart from the anti-phospho-S392 antibody, which gave a very weak signal also for non-irradiated mESCs, none of the antibodies recognised p53 from undamaged cells. p53 activity, however, was compromised in mESCs under normal culture conditions. Thus, p53 from mESCs and from differentiated cells may possess post-translational modifications that control their function under normal culture conditions and these modifications may be different from those after genotoxic stress. To investigate this possibility, we performed two-dimensional western blotting. In agreement with earlier findings, we found that p53 is an acidic protein.^14^ The majority of p53 from ESCs and from differentiated cells migrated with a pI between 4.8 and 5.2, whereas a minor amount migrated with a pI between 5.3 and 6.4. Interestingly, mESCs also harboured a fraction of p53 that migrated with a pI between 6.4 and 8.2 that was not present in differentiated cells. Thus, a part of p53 was indeed different between mESCs and differentiated cells (Figure 3b). To see whether this fraction of p53 is just unphosphorylated protein, we treated the cell lysate with lambda phosphatase. For control, we irradiated cells with gamma rays resulting in the phosphorylation of p53 at serine 15. This phosphorylation was largely removed upon phosphatase treatment (Supplementary Figure S4A). When we treated the lysate of non-irradiated stem cells and differentiated cells with lambda phosphatase under the same condition, we observed a fraction of p53 from stem cells with a pI beyond 8.6 that was not detectable in stem cells without phosphatase treatment (Figure 3c), showing that p53 in stem cells is constitutively phosphorylated, at least to some extent. The charge of p53 from differentiated cells was also altered upon phosphatase treatment although no p53 with a pI beyond 8.6 was detected, indicating that additional modifications with a negative charge may exist in differentiated cells. Literally, p53 from differentiated cells treated with phosphatase resembled untreated p53 from stem cells (Figure 3c). Another post-translational modification that affects the charge of a protein is acetylation. To see whether p53 from stem cells is acetylated, we treated stem cells and differentiated cells with the HDAC inhibitors trichostatin A and nicotinamide prior to cell lysis. For control, we analysed an aliquot of the treated cells for p53 acetylation at lysine 379. Treatment of stem cells and differentiated cells with trichostatin A/nicotinamide resulted in strong acetylation of p53 and an increase in overall p53 abundance (Supplementary Figure S4B). This treatment furthermore strongly increased the negative charge of p53 from stem cells resulting in the complete removal of the fraction of p53 with a neutral pI (Figure 3d). In differentiated cells, the fraction of p53 with a pI between 4.6 and 5.2 was also increased. The effect was, however, not as strong as in stem cells (Figure 3d).

### p53 is associated with MdmX in stem cells

**Figure 4:**
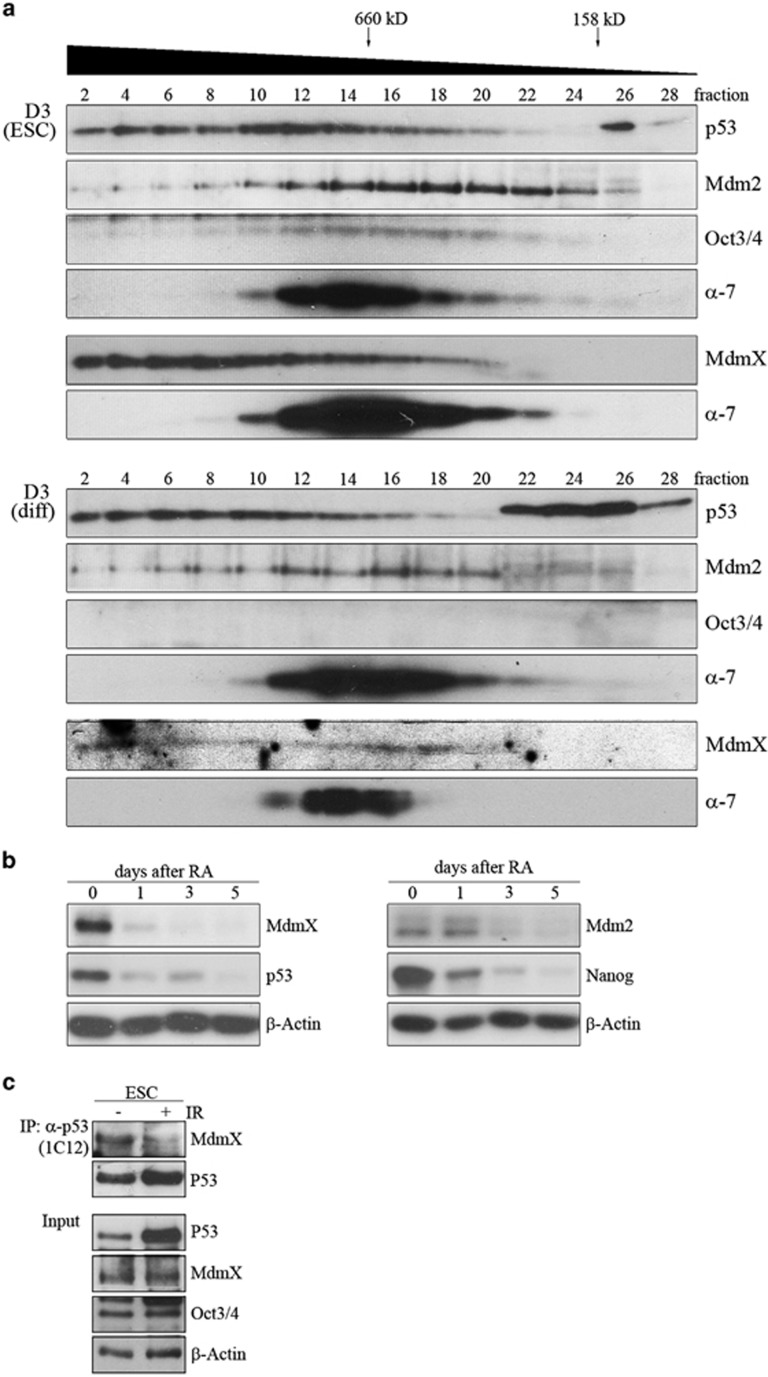
p53 is associated with MdmX in mESCs. (**a**) D3 cells and D3 cells that had been differentiated for 7 days with retinoic acid (D3 diff.) were lysed. After sucrose density centrifugation of the lysates, 28 fractions were collected and analysed for the abundance of p53, Mdm2 and MdmX. Abundance of Oct3/4 was determined to control for stemness and hybridisation with *α*-7 was performed for internal control. (**b**) D3 cells were treated with retinoic acid (RA) for differentiation. At the indicated number of days after initiation of differentiation, cells were lysed and abundance of MdmX, p53 and Mdm2 was monitored by western blotting. Abundance of Nanog was determined to control for stemness and of *β*-Actin for loading control. (**c**) D3 cells were irradiated with 7 Gray and lysed 3 h after irradiation. p53 was precipitated and associated MdmX was monitored by western blotting. An aliquot of the cellular lysate was used to monitor the abundance of p53 and MdmX in the whole cellular lysate. Hybridisation with Oct3/4 was performed to control for stemness and with *β*-Actin for loading control

As a fraction of p53 with a more neutral pI existed exclusively in mESCs, we wondered whether this affected the association of p53 with other proteins and performed sucrose density centrifugation. Interestingly, most p53 was found in complexes larger than 500 kD and hardly any p53 protein existed as monomers or dimers. However, we could not see a difference between mESCs and differentiated cells (Figure 4a; the strong signals in fraction 26–28 of mESCs and fraction 22–28 of differentiated cells is unspecific as it was also seen in p53^−/−^ cells).

p53 activity is mainly inhibited by Mdm2 and MdmX (Mdm4).^3^ We therefore wondered whether binding of p53 to Mdm2 and MdmX is altered in mESCs and differentiated cells. If Mdm2 and MdmX are associated with p53, they should co-elute with p53 from sucrose gradients. Indeed, the elution profile of p53 and MdmX from mESCs and differentiated cells was superimposable, yet the signal for MdmX was considerably weaker in differentiated cells (Figure 4a). Surprisingly, the majority of Mdm2 (Figure 4a) was present in complexes that were much smaller than the majority of the complexes containing p53. From the 28 fractions only 6 (fraction 12-18) contained larger amounts of both, p53 and Mdm2. However, there was no discernible difference in the distribution of Mdm2 between mESCs and differentiated cells, yet the signal for Mdm2 was weaker in differentiated cells (Figure 4a).

Our results suggested that the amount of Mdm2 and MdmX might be decreased during differentiation. Indeed, MdmX levels were very high in stem cells and decreased rapidly during differentiation, simultaneously with p53 and the stem cell marker Nanog (Figure 4b). Abundance of Mdm2 also decreased during differentiation, but the decrease was slower and weaker (Figure 4b).

To see whether MdmX associates with p53, we performed co-immunoprecipitations. For control, we irradiated cells, a treatment that has been shown to downregulate MdmX.^15^ MdmX indeed co-precipitated with p53 from mESCs. After irradiation, this interaction was strongly reduced, showing the specificity of the co-precipitation (Figure 4c).

**Figure 5:**
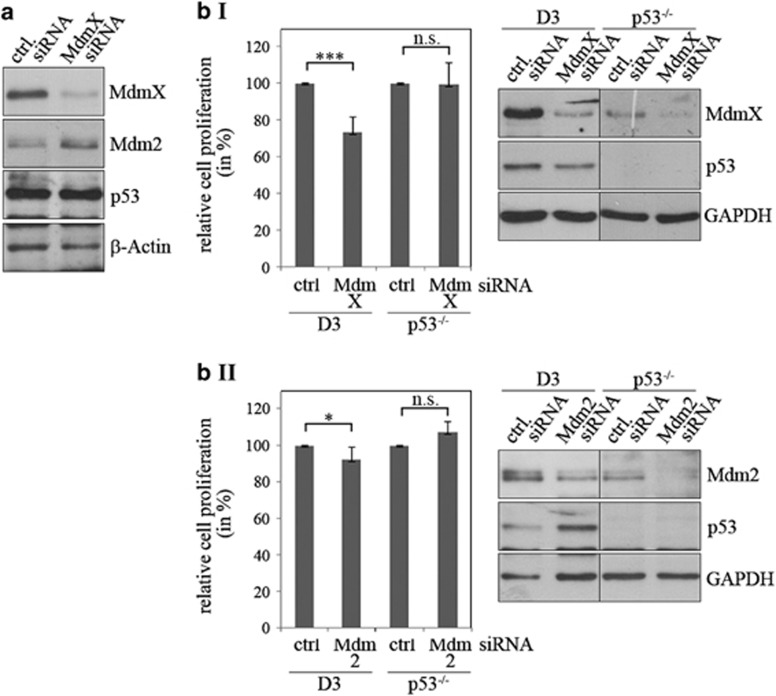
p53's activity is controlled by MdmX in mESCs. (**a**) D3 cells were transfected with a siRNA that was directed against mdmX or with a control siRNA. Forty-eight hours after transfection, cells were lysed. Abundance of MdmX, Mdm2 and p53 was monitored by western blotting. Hybridisation with *β*-Actin was used for loading control. (**b**) Wild-type D3 cells and D3 cells with a genetic deletion of p53 (p53^−/−^) were transfected in duplicates with a siRNA directed against mdmX (**b I**), mdm2 (**b II**) or with a control siRNA. Forty-eight hours after transfection, one of the duplicates was lysed to control for the downregulation by western blotting. The second duplicate was used to monitor relative cell numbers by MTT assay. The graph shows mean values and standard deviation of the relative cell numbers of three (downregulation of Mdm2 in p53^−/−^ cells) to five (downregulation of MdmX) independent experiments. Relative cell numbers of cells that had been transfected with control siRNA were set to 100%. *P<0.05; ***P<0.005

To see whether MdmX indeed inhibits p53 activity in mESCs, we downregulated MdmX. Downregulation of MdmX indeed increased abundance of the p53-transcriptional target Mdm2 (Figure 5a). Downregulation of MdmX furthermore reduced cell proliferation in p53-positive mESCs to less than 75%, whereas no difference in cell numbers was discernible in p53-negative mESCs (Figure 5b.I). Surprisingly, under the same conditions, downregulation of Mdm2 reduced cell proliferation only by approximately 7% (Figure 5b.II).

### Wild-type p53 controls a similar set of target genes in mESCs as mutant p53 in differentiated cells

**Figure 6:**
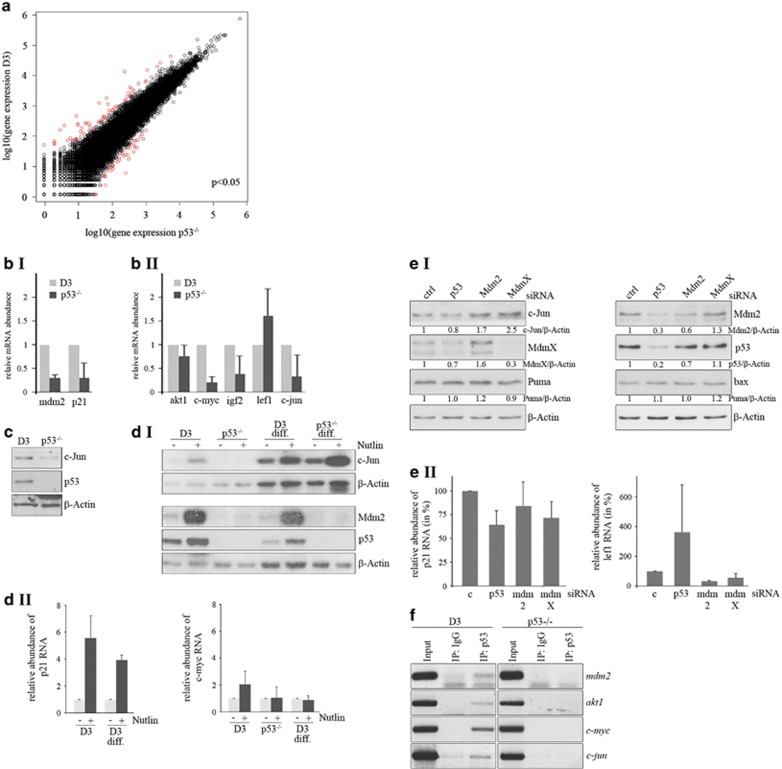
p53 is active in stem cells. (**a**) Transcriptome analysis of wild-type (D3) and p53-negative (p53^−/−^) D3 cells by RNAseq. The graph shows plotting of the log10 of gene expression under both conditions. Genes with a fold change ⩾2 are shown in red. (**b**) Wild-type D3 cells and D3 cells with a genetic deletion of p53 (p53^−/−^) were lysed. RNA was prepared, transcribed into cDNA and subjected to qRT-PCR. Abundance of specific cDNAs was normalised by determining the abundance of the housekeeping gene RibPO. Mean values and error bars of two independent experiments were calculated and plotted. Relative abundance of the specific RNA in D3 cells was set to 1. (**c**) Wild-type D3 cells and D3 cells with a genetic deletion of p53 (p53^−/−^) were lysed. Abundance of c-Jun and p53 were monitored by western blotting. Hybridisation with *β*-Actin was carried out for loading control. (**d**) D3 cells, their p53-negative counterpart (p53^−/−^) and D3 and p53^−/−^ cells that had been differentiated with retinoic acid for seven days (D3 diff., p53^−/−^ diff.) were treated with 5 *μ*M nutlin for 32 h. A part of the cells was lysed and abundance of c-Jun, Mdm2 and p53 was determined by western blotting (**d I**). From the remaining cells, RNA was prepared and relative abundance of p21 and c-myc was determined by qRT-PCR. Abundance of specific cDNAs was normalised by the abundance of the housekeeping gene RibPO. The graph shows mean values and error bars of two independent experiments. Relative abundance of the specific RNA in mock-treated cells was set to 1 (**d II**). (**e**) D3 cells were transfected with a siRNA targeted against p53, Mdm2 or MdmX, or with a control siRNA. After harvest, the cells were separated into two parts. One of the samples was analysed by western blotting to determine the abundance of c-Jun, Puma and bax and of p53, Mdm2 and MdmX (**e I**). From the second part, RNA was prepared and the abundance of p21 and lef1 was determined by qRT-PCR. The graph shows mean values and error bars of two independent experiments. Relative abundance of p21 and lef1 RNA in control siRNA-transfected cells was set to 100% (**e II**). (**f**) D3 cells and their p53-deficient counterpart (p53^−/−^) were lysed. p53 was precipitated (IP: p53) and associated *c-myc*, *c-jun*, *akt-1* and *mdm2* DNA was monitored by PCR. Precipitation with IgG and total cell lysate (Input) were used for control

As p53 levels are high in mESCs (Supplementary Figure S1A),^8^ we reasoned that p53 may fulfil an important function in these cells which could be different to its function in differentiated cells. We therefore analysed the transcriptomes of unstimulated p53^−/−^ and p53^+/+^ mESCs of the same genetic background. We observed a considerable overlap of the transcriptome of p53^+/+^ and p53^−/−^ ESCs, but there were also some clear differences (Figure 6a). Among the genes that were induced by p53 in mESCs were *fosb*, *mdm2*, *cdkn1* (p21), *akt1*, c-*myc*, *c-jun, igf2, cyclin D1 and cyclin D2*. Remarkably, these genes are also induced by mutant p53 in tumour cells.^6, 7^ To consolidate the data from the RNA sequencing, we performed qRT-PCR of some of the genes. In accordance with data from differentiated cells,^1^ *mdm2* and *cdkn1 (p21)* expression was higher in p53-positive mESCs (Figure 6b.I). Most interestingly, expression of *akt1, c-myc*, *c-jun* or *igf2* was also significantly higher in p53-positive mESCs than in p53-negative mESCs (Figure 6b.II). These increases were not due to an overall increase in gene expression as, for example, expression of *lef1* was reduced in wild-type ESCs compared with p53^−/−^ mESCs (Figure 6b.II). This induction of proto-oncogenes by wild-type p53 is also translated into protein, as mESCs with wild-type p53 possessed more c-Jun protein than p53^−/−^ mESCs (Figure 6c). Treatment of stem cells with nutlin not only resulted in a p53-dependent induction of the classical p53 target genes Mdm2 and p21, but also of c-Jun and c-myc in stem cells (Figure 6d,Supplementary Figure S5). This induction of c-Jun and c-myc was not seen in p53-negative stem cells. Although nutlin clearly failed to increase c-myc RNA levels in differentiated cells, it induced c-Jun in differentiated cells. This induction, yet, also occurred in p53-negative differentiated cells (Figure 6d). Downregulation of Mdm2 or MdmX increased abundance of c-Jun, whereas downregulation of p53 by siRNA reduced its abundance (Figure 6e) further supporting that p53 controls *c-jun* expression in stem cells. In consistency, lef1, which we found to be downregulated by p53 in stem cells was induced after downregulation of p53 and further reduced after downregulation of Mdm2 and MdmX (Figure 6e). In contrast, p21, although reduced after downregulation of p53, was not increased after downregulation of Mdm2 and MdmX (Figure 6e), suggesting that further conditions must be met to allow induction of p21 by p53. In line with the regulation of akt1, c-myc and c-jun by wild-type p53 in stem cells, we found p53 associated with their promoters. Importantly, we could precipitate *akt1*, *c-myc* and *c-jun* DNA with an anti-p53 antibody only from lysates of p53-positive stem cells, but not from p53-negative stem cells or differentiated cells (Figure 6f,Supplementary Figure S5B).

As p53 is activated in response to DNA damage,^16^ we wondered whether p53 induces c-myc or c-jun in stem cells also in response to DNA damage. When we treated stem cells with the topoisomerase inhibitor etoposide to induce DNA strand breaks, we observed a strong induction of p53 and a p53-dependent increase in Mdm2 and p21 (Supplementary Figure S5C). We also observed induction of c-Jun and downregulation of lef1 in response to DNA damage. However, c-Jun was also induced and lef1 also downregulated in p53-negative cells, making it questionable whether p53 contributed to their regulation after DNA damage (Supplementary Figure S5C). In contrast, c-myc, which was normally induced by p53 in stem cells was reduced in response to etoposide treatment and this reduction was p53 independent. Thus, it is rather unlikely that p53 controls its 'stem cell-specific target genes' also in response to DNA damage.

Overall, these results demonstrate that p53 alters the transcriptional program in mESCs. Most remarkably, p53 controls expression of a different set of genes in mESCs than in differentiated cells and, most strikingly, several of these genes are also controlled by mutant p53 in tumour cells.

## Discussion

### p53 is nuclear in mESCs

By using different antibodies, we show that the majority of p53 is nuclear in mESCs, which is in contrast to previous reports who described p53 as a cytoplasmic protein in mESCs.^9, 10, 17^ The disagreement is most likely due to insufficient specificity of the antibodies used in previous studies. The antibodies Pab421 or Pab246 gave signals in the cytoplasm of mESCs both after immunostaining and cell fractionation/western blotting. However, in all cases, we observed a similar signal also in p53-negative mESCs, indicating that these antibodies recognised a protein different from p53. The antibodies 1C12 and CM5 that recognised p53 specifically showed that the majority of p53 was in the nucleus of mESCs. In agreement with Li and co-workers,^18^ we found that most of the p53 in the nucleus was associated with DNA. After leptomycin B treatment, p53 accumulated in the nucleus, according to its nuclear localisation in mESCs and its CRM1-mediated shuttling between nucleus and cytoplasm.^11^ Surprisingly, p53 also accumulated in the cytoplasm after treatment with leptomycin B, indicating that proteins that degrade p53 may also shuttle between nucleus and cytoplasm.

### p53 activity in mESCs is controlled by MdmX

When p53 is not properly controlled, it induces cell cycle arrest and cell death. The most well-known regulators of p53 are Mdm2 and MdmX that both associate with the transactivation domain of p53.^3^ The most striking difference between Mdm2 and MdmX is that Mdm2 has a functional RING-domain and thus targets p53 for degradation whereas MdmX does not.^3^ Although Mdm2 is ubiquitously expressed, MdmX is hardly detectable in most untransformed tissues. Surprisingly, MdmX was highly expressed in mESCs. Moreover, MdmX co-fractionated with p53 from sucrose gradients, co-precipitated with p53 from mESC lysate and controled p53's transcriptional and anti-proliferative function in mESCs. Previously, it was shown that MdmX controls p53 activity during differentiation.^19^ With this study, we extend this activity to mESCs. Nevertheless, although MdmX is most likely an important factor for controlling p53 activity in stem cells, it may not be the only one. TRIM25 for example, a member of the TRIM protein superfamily that controls p53 activity (Zhang *et al.*, accepted), is also more abundant in stem cells than in differentiated cells (Ping Zhang, unpublished results) and further proteins with such properties may exist. Thus, rather than relying on one single protein for controlling p53 activity, mESCs may have a set of proteins with overlapping function to ensure that p53 is kept under control.

### p53 alters the transcriptional program of stem cells

ESCs have high amounts of p53.^8^ As p53 is an anti-proliferative protein, it was previously thought that p53 is inactive in stem cells. Indeed, p53's anti-proliferative activity was compromised in mESCs. However, by RNA sequencing, we found that the transcriptional profile of p53-positive and p53-negative mESCs is different demonstrating that p53 influences the transcriptional program in mESCs. Whether p53 activates gene transcription directly or whether its influence is more indirect remains to be determined. As p53-negative stem cells had a different transcriptional pattern than wild-type stem cells, we asked whether some of the changes might be adaptations to the loss of p53. Yet when we downregulated p53, we observed similar changes as in p53-negative stem cells.

Intriguingly, many genes that were upregulated in mESCs with wild-type p53 are induced by mutant p53 in tumour cells, implying that wild-type p53 in stem cells may have acquired properties of tumour-derived mutant p53. Interestingly, p53 from stem cells associated with the same region of the *c-myc* promoter that is occupied by mutant p53 in differentiated cells.^20^ Furthermore, p53 associated with GC-rich regions around the transcriptional start site of *c-jun* and *akt-1* in mESCs, a property that has been described for mutant p53 in differentiated cells.^21^ Mutant p53 from tumour cells has frequently an altered conformation that allows novel protein–protein interactions.^6, 7^ We think that a similar mechanism may apply for p53 in stem cells, particularly as we could not find a consensus binding site for p53 in the 'novel' p53 target genes. Whether p53 from stem cells is undergoing similar protein–protein interactions in mESCs as mutant p53 in differentiated cells remains to be determined. Interestingly, we found a fraction of p53 with a neutral pI in mESCs that is absent in differentiated cells indicating that p53 from mESCs and differentiated cells are differently modified. These modifications may enable p53 to control a different set of target genes in stem cells and differentiated cells.

As p53 is activated in response to cellular stress,^3, 17^ we wondered whether p53 would induce these 'stem cell-specific' target genes also after DNA damage. However, although the classical p53 target genes p21 and mdm2 were upregulated after treatment of stem cells with etoposide, expression of *c-myc* was strongly reduced both in p53-positive and p53-negative stem cells. c-Jun was induced in p53-positive and p53-negative stem cells and lef1 was reduced in p53-positive and p53-negative stem cells, making it unlikely that p53 controls its stem cell-specific targets in response to DNA damage.

## Materials and methods

### Cell lines and their treatment

D3 cells were cultured in Glutamax-I medium (Life Technologies, Darmstadt, Germany) supplemented with 15% fetal bovine serum, 1 × non-essential amino acids, 0.1 mM *β*-mercaptoethanol, 1% penicillin/streptomycin and 1000 units/ml LIF. Mouse embryonic fibroblasts (MEFs) that had been irradiated with 6.3 Gray served as feeder cells. p53-negative stem cells (p53^−/−^) were cultured in Glutamax-I medium (Life Technologies) supplemented with 15% fetal bovine serum, 1 × non-essential amino acids, 0.1 mM *β*-mercaptoethanol, 1% penicillin/streptomycin and 1000 units/ml LIF. MEF cells that had been irradiated with 6.3 Gray served as feeder cells. MEFs were cultured in Dulbecco's modified Eagle medium (Life Technologies) supplemented with 10% fetal bovine serum and 1% penicillin/streptomycin. For differentiation, cells were cultured in Dulbecco's modified Eagle medium-GlutaMAX-I medium supplemented with 10% fetal bovine serum, 1 × non-essential amino acids, 0.1 mM *β*-mercaptoethanol, 1% penicillin/streptomycin and 1 *μ*M of all-*trans*-retinoic acid (Sigma-Aldrich, Taufkirchen, Germany) for 7 days on culture dishes coated with 0.1% gelatin.

Cells were irradiated with a ^60^Co-gamma source at a dose rate of 0.5 Gray per minute in cell culture medium. Etoposide was used at a final concentration of 50 *μ*M. Leptomycin B (Sigma-Aldrich) was added to a final concentration of 2 *μ*M after serum starvation and incubated for 16 h. Trichostatin A was used at a final concentration of 2.5 mM and nicotinamide at a final concentration of 7.5 mM.

### Antibodies and siRNAs

The anti-p53 antibodies Pab421, Pab246, CM5 and 1C12 were purchased from Millipore (Darmstadt, Germany), Vector laboratories (Burlingame, CA, USA) and Cell signaling (Danvers, MA, USA), respectively. From Santa Cruz (Dallas, TX, USA), we obtained the antibodies against Oct-4 (C-10) Nanog (C-4), c-Jun (H79) and PCNA (PC10). Antibodies against acetylated and phosphorylated p53 (K379, S6, S15, S392) were from Cell signaling. Antibodies against GAPDH (6C5), PARC (PO69) and *α*-7 (MCP72) were from Hytest (Turku, Finland), BioLegend (San Diego, CA, USA) and Enzo (Loerrach, Germany), respectively. Antibodies against *β*-Actin and Histone H3 were purchased from Abcam (Cambridge, UK) and the antibody against MdmX (MDMX82) was from Sigma-Aldrich.

siRNAs were purchased from Eurofins (Ebersberg, Germany). Sequences are available on request. siRNA transfections were performed using Macsfectin (Miltenyi Biotec, Bergisch-Gladbach, Germany) following the manufacturer's instructions.

### Cell lysis and western blotting

Cell lysis and 1-D western blotting was performed as described.^22^ For 2-D western blotting, cells were suspended in Urea Lysis buffer (8.5 M Urea, 4% CHAPS, 50 mM Tris pH8.0, 1 mM PMSF) and disrupted by sonication at Amp 50 for 10 s. The lysate was cleared by centrifugation for 20 min at 15000 r.p.m. Protein (0.5–1 mg) was diluted with an equal volume of 2 × IEF sample buffer (5 M Urea, 2 M Thio-urea, 65 mM CHAPS, 4 mM tributylphosphin and 1% Carrier-ampholyte pH 3-10 (Serva, Heidelberg, Germany)). The volume was adjusted to 335 *μ*l with Rehydration buffer (5 M Urea, 2 M Thio-urea, 65 mM CHAPS, 2 mM tributylphosphin, 0.5% Carrier-ampholyte pH 3-10). 5 μl 0.8% Commassie Brilliant Blue was added. This solution was used to hydrate an 18 cm-Immobiline DryStrip pH 3-11 (GE Healthcare, Munich, Germany). Isoelectric focusing was performed using an Ettan IPGphor II Isoelectric Focusing system (GE Healthcare) at 25 ^o^C and 200 V for 3.5 h, at 500 V for 3.5 h, at 1000 V for 3.5 h, at a gradient ramp up to 8000 V for 1 h and at 8000 V for 11 h. The IEF-gel was equilibrated in equilibration buffer (50 mM Tris pH 8.8, 6 M urea, 30% Glycerol, 2% SDS, 65 mM DTT) for 15 min and alkylated for 15 min in alkylation buffer (50 mM Tris pH 8.8, 6 M urea, 30% Glycerol, 2% SDS, 20 mg/ml iodoacetamide, 0.03% Commassie Brilliant Blue). The second dimension was performed in a Bio-Rad (Munich, Germany) SDS-PAGE 1D cell using 10 or 12.5% SDS polyacrylamide gels. After electrophoresis, the gel was blotted and p53 and PCNA proteins were monitored by immunodetection.

For phosphatase treatment, cells were suspended in phosphatase buffer and lysed by mild sonication. Two hundred units lambda phosphatase were added per 100 *μ*g protein and incubated for 30 min at 30 °C. The proteins were TCA-precipitated and suspended in Urea lysis buffer.

For cell fractionation, the cellular pellet was suspended in 4 packed cell volumes lysis buffer (10 mM HEPES pH7.4, 50 mM NaCl, 0.5 M sucrose, 0.5% Triton x-100, 1 mM PMSF), incubated for 5 min on ice and centrifuged at 1000 r.p.m. for 10 min. The cytoplasmic fraction (supernatant) was transferred to a new tube. The pellet was washed twice with cold PBS, suspended in the same volume lysis buffer as the cells at the beginning of the procedure and disrupted by sonication.

For DNAse treatment, DNAse (Pierce Universal Nuclease for Cell lysis, Thermo Fisher Scientific, Bonn, Germany) was added to the nuclear lysate and incubated for 1 h on ice.

### Immunofluorescence staining

ES cells were grown on gelatinised cover slips. After washing two times with ice-cold PBS, cells were fixed with ice-cold acetone/methanol (1 : 1) for 8 min on ice. Then the cover slips were washed three times with PBS and blocked for 30 min with blocking buffer (1% bovine serum albumin, 1% goat serum in PBS). After blocking, cells were incubated overnight with the first antibody. The next day, the cover slips were washed three times with PBS and incubated for 30 min at room temperature in the dark with an antibody directed against mouse IgG coupled with Alexa-Fluor-488 (Life Technologies) or with an antibody directed against rabbit IgG coupled to Alexa-Fluor-546 (Life Technologies) together with Draq5 (Biostatus Limited, Shepshed, UK), all diluted 1 : 1000 in blocking buffer. Finally, the cover slips were washed three times with PBS, mounted with Hydromount on microscope slides and analysed by microscopy.

### Immunoprecipitation

Cells were washed twice with ice-cold PBS and lysed in Co-IP lysis buffer (10 mM HEPES, pH 7.4, 50 mM NaCl, 0.5 M sucrose, 0.5%Triton x-100, 1 mM PMSF, 1 × Phosphostop (Roche, Mannheim, Germany), 200 U/ml DNAse (Universal Nuclease for cell lysis; Pierce). One milligram of protein was added to protein A-agarose (Pierce) pre-coupled with the anti-p53 antibody 1C12. After 2 h incubation on a rotating wheel at 4 °C, the precipitates were washed three times with Co-IP wash buffer (50 mM Tris, pH 7.5, 1 mM EDTA, 100 mM NaCl, 0.1% Triton x-100, 5% glycerol). Thirty microlitres of 2 × SDS sample buffer (4% sodium dodecyl sulphate, 0.16 M Tris pH 6.8, 20% glycerol, 4% *β*-mercaptoethanol, 0.002% bromphenol blue) were added to the precipitates prior to heat-denaturation and loading onto a 10% SDS-PAGE gel.

### Sucrose gradient centrifugation

Cells were washed twice with ice-cold PBS, scraped into PBS and collected by centrifugation. Cells were suspended in (50 mM Tris pH 7.4, 20 mM NaCl, 10 mM MgCl_2_, 0.5% NP40, 5 mM ATP, 1 mM DTT, 1 mM PMSF, 40 U/ml DNAse (Universal Nuclease for cell lysis; Pierce), 10 mM N-ethylmaleimide, 10 mM 1,10-phenanthtroline and 1 × Phosphostop), pushed three times through a 26G needle and incubated on ice for 30 min. The protein extract was cleared by centrifugation at 16 000 × *g* at 4 °C for 15 min, loaded onto a 10–40% sucrose gradient and centrifuged at 100 000 × *g* at 4 °C for 18 h. Fractions were collected and analysed by western blotting.

### Proliferation assays

Cells were plated on gelatin-coated (stem cells) or non-coated (mouse embryonic fibroblasts) plates and counted each day. For MTT assays, *3*-[4,5-dimethylthiazol-2-yl]-2,5-diphenyltetrazolium bromide was added to a final concentration of 0.2 mg/ml and incubated for 4 h. The medium was removed and cells and the formazan salt were solubilised in 1.5 ml isopropanol. One hundred and fifty microlitres of this solution were transferred to a microplate and the absorbance was determined at *λ*550 nm by a microplate reader.

### RNA sequencing

Total RNA was prepared using the TRIzol reagent (Life Technologies) according to the manufacturer's instructions. Extracted total RNA samples were tested on RNA nanochips (Bioanalyser 2100, Agilent, Santa Clara, CA, USA), and showed no sign of degradation (RNA index number>8). Sequencing libraries were generated from 1 *μ*g of RNA samples with the TruSeq stranded mRNA kit (Illumina, San Diego, CA, USA). Size and quality of sequencing libraries were determined on DNA-chip (Bioanalyser 2100, Agilent). Multiplexed samples at 7pM were loaded on a single lane Illumina Flow cell v3. Paired end reads (2 × 50 nucleotides) were obtained on a Hiseq1000 using SBS v3 kits (Illumina). Cluster detection and base calling were performed using RTAv1.13 and quality of reads assessed with CASAVA v1.8.1 (Illumina). The sequencing resulted in >67 million pairs of 50-nt long reads per sample with a mean Phred quality score>35 (Supplementary Table 1). The reads were mapped against the mouse genome (M37) using tophat version 1.4.1^23^ and bowtie 0.12.7 with the options —butterfly-search —coverage-search —microexon-search -a 5 -p 5 —library-type fr-unstranded and using known exon junctions (Ensembl release 67). The mean distance and standard deviation between read pairs were obtained from CASAVA. Gene expression was determined with HTSeq version 0.5.3p3^24^ by counting for each gene the number of reads that overlapped with the annotation location obtained from Ensembl release 67. Differential expression was calculated using the *R* package DESeq.^24^

### qRT-polymerase chain reaction

Total RNA was prepared from cells using the RNeasy kit (Qiagen, Hilden, Germany) according to the manufacturer's recommendation. The RNA was treated with DNaseI to remove residual genomic DNA and transcribed into cDNA using random primers and RevertAid H MinusM-MuLV reverse transcriptase (Fermentas, St. Leon-Rot, Germany). Real-time PCR was performed in duplicates with a SYBR Green PCR mixture (Qiagen/Promega, Mannheim, Germany). The cDNA was denatured for 15 min at 95 °C followed by 40 cycles of 95 °C for 15 s and 50 °C for 1 min using the 7000 ABI sequence detection system and gene specific primers. The signals were normalised to the signals for the housekeeping gene RibPO (34B4). See Supplementary Table 3 for primer sequences.

### Chromatin immunoprecipitation

Proteins and DNA were cross-linked by incubation with formaldehyde (1% f.c.) for 10 min at room temperature. The reaction was stopped by incubation with glycine (0.125 M f.c.) for 5 min at room temperature. The media was removed and the plates were washed three times with ice-cold PBS. The cells were scraped from the culture dish, collected by centrifugation and washed with PBS containing 1 mM PMSF. Cells were suspended in 3 × volumes of lysis buffer (5 mM HEPES pH 8, 85 mM KCL, 0.5% NP40, 1 mM PMSF, 1 *μ*g/ml aproptinin, 1 *μ*g leupeptin) and incubated for 10 min. The nuclei were pelleted for 5 min at 5000 r.p.m. and 4 °C, suspended in nuclei lysis buffer (50 mM Tris pH 8, 10 mM EDTA 8.1, 1% SDS, 1 mM PMSF, 1 *μ*g/ml aproptinin, 1 *μ*g/ml leupeptin) and incubated for 10 min on ice. The lysate was sonicated to achieve an average length of the chromatin of about 400 bp and cleared by centrifugation at 13 000 r.p.m. for 10 min at 4 °C. The supernatant was diluted five-fold in ChIP dilution buffer (0.01% SDS, 1.1% Triton X 100, 1.2 mM EDTA pH 8.1, 16.7 mM Tris-Cl pH 8.0, 167 mM NaCl, 1 mM PMSF, 1 *μ*g/ml aproptinin, 1 *μ*g/ml leupeptin). The samples were pre-cleared with a mixture of sonicated salmon sperm DNA, BSA and protein A agarose for 30 min at 4 °C. The beads were pelleted and the supernatant was transferred to a new tube. Ten percent of the supernatant was kept for input control. The remaining lysate was divided into two parts. To one part, IgG was added, and to the other part, the anti-p53 antibody CM5. The samples were incubated at 4 °C overnight with end-over-end rotation. Then, a slurry of sonicated salmon sperm DNA, protein A agarose and BSA was added and incubated for 1 h at 4 °C with end-over-end rotation. The precipitates were collected by centrifugation at 7200 r.p.m. and 4 °C for 3 min and consecutively washed once with low salt wash buffer (0.1% SDS, 1% Triton X 100, 2 mM EDTA pH (8.1, 20 mM Tris-Cl, pH 8.0), twice with high salt wash buffer (0.1% SDS, 1% Triton X 100, 2 mM EDTA, pH 8.1, 20 mM Tris-Cl, pH 8.0, 500 mM NaCl), once with LiCl wash buffer (10 mM Tris-Cl pH 8.0, 250 mM LiCl, 1% NP40, 1% deoxycholic acid, 1 mM EDTA pH 8.1) and twice with TE buffer (10 mM Tris pH 8.0, 1 mM EDTA pH 8.1). Any traces of buffer were removed and the antibody/protein/DNA complexes were eluted twice with freshly made elution buffer (100 mM NaHCO_3_, 1% SDS). The supernatants were combined and centrifuged to remove any traces of protein A agarose. The crosslinks were removed from the precipitated samples and input controls by incubation with RNAse A in the presence of 0.3 M NaCl (f.c.) at 65 °C overnight. Proteins and DNA were precipitated by addition of 2½ volumes ethanol at −80 °C for 1 h and collected by centrifugation. The pellets were suspended in 0.01 M EDTA pH 8.1, 0.04 M Tris-Cl pH 6.5 and 150 *μ*g/ml proteinase K and incubated for 2 h at 45 °C. 175 *μ*l TE buffer were added and the samples were extracted once with phenol/chloroform/isoamyl alcohol and once with chloroform isoamyl alcohol. The samples were adjusted to 0.85 M NaCl. 5 *μ*g tRNA and Roti-Pink (Roth, Karlsruhe, Germany) and 800 *μ*l ethanol were added and the DNA was precipitated at −20 °C overnight. The DNA was pelleted by centrifugation, diluted with H_2_O and analysed by PCR using 1 *μ*l of each primer (50 *μ*g/*μ*l), 0.5 *μ*l dNTPs (10 mM) and GoTaq polymerase (Promega). See supplementary table 2 for primer sequence and cycle conditions.

## Abbreviations

MdmX: mouse double minute X
Mdm2: mouse double minute 2
mESCs: murine embryonic stem cells
pI: isoelectric point
GAPDH: glyceraldehyde 3-phosphatdehydogenase
kD: kilo dalton
LMB: leptomycin B
h: hour
CRM1: chromosome region maintenance 1
MEFs: murine embryonic fibroblasts
MTT: *3*-[4,5-dimethylthiazol-2-yl]-2,5-diphenyltetrazolium Bromide
K379: lysine 379
S6: serine 6
S15: serine 15
S392: serine 392
HDAC: histone deacetylase
TSA: trichostatin A
NA: nicotinamide
Cdkn1: cyclin-dependent kinase inhibitor 1
Igf2: insulin-like growth factor 2
LIF: leukemia inhibitory factor
μM: micromolar
mM: millimolar
RNA: ribonucleic acid
siRNA: small interfering RNA
CHAPS: 3-[(3-cholamidopropyl)dimethylammonio]-1-propanesulfonate
PMSF: phenylmethylsulfonylflurorid
min: minute(s)
rpm: rounds per minute
μg: microgram
mg: milligram
IEF: isoelectric focusing
μl: microliter
ml: milliliter
°C: degree Celsius
V: volt
cm: centimeter
SDS: sodiumdodecylsulfate
DTT: dithiothreitol
HEPES: (4-(2-hydroxyethyl)-1-piperazineethanesulfonic acid
NaCl: sodiumchlorid
PBS: phosphate-buffered-saline
IgG: immunoglobulin G
Co-IP: co-immunoprecipitation
PAGE: polyacrylamide gel electrophoresis
MgCl_2_: magnesium chloride
NP40: nonidet P40
ATP: adenosine-triphosphate
G: gauge
l: lambda
mRNA: messenger ribonucleic acid
nt: nucleotide
qRT-PCR: quantitative real time polymerase chain reaction
cDNA: copy DNA
MuLV: murine leukemia virus
RibPO: riboprotein
f.c.: final concentration
KCL: potassium chloride
EDTA: ethylenediaminetetraacetic acid
ChIP: chromatin-immunoprecipitation
BSA: bovine serum albumin
LiCl: lithium chloride
TE: tris-EDTA
M: molar
tRNA: transfer RNA
dNTPs: deoxynucleotides
WCL: whole cell lysate
ECL: enhanced chemiluminescence
PCNA: proliferating cell nuclear antigen
TCA: trichloracetic acid
Oct3/4: octamer binding transcription factor3/4
IP: immunoprecipitation
