# Supplementary figures and images for: p53 is active in murine stem cells and alters the transcriptome in a manner that is reminiscent of mutant p53
Source: Cell Death Dis. 2015 Feb 26;6(2):e1662–. doi: 10.1038/cddis.2015.33 (PMC4669809; doi:10.1038/cddis.2015.33)

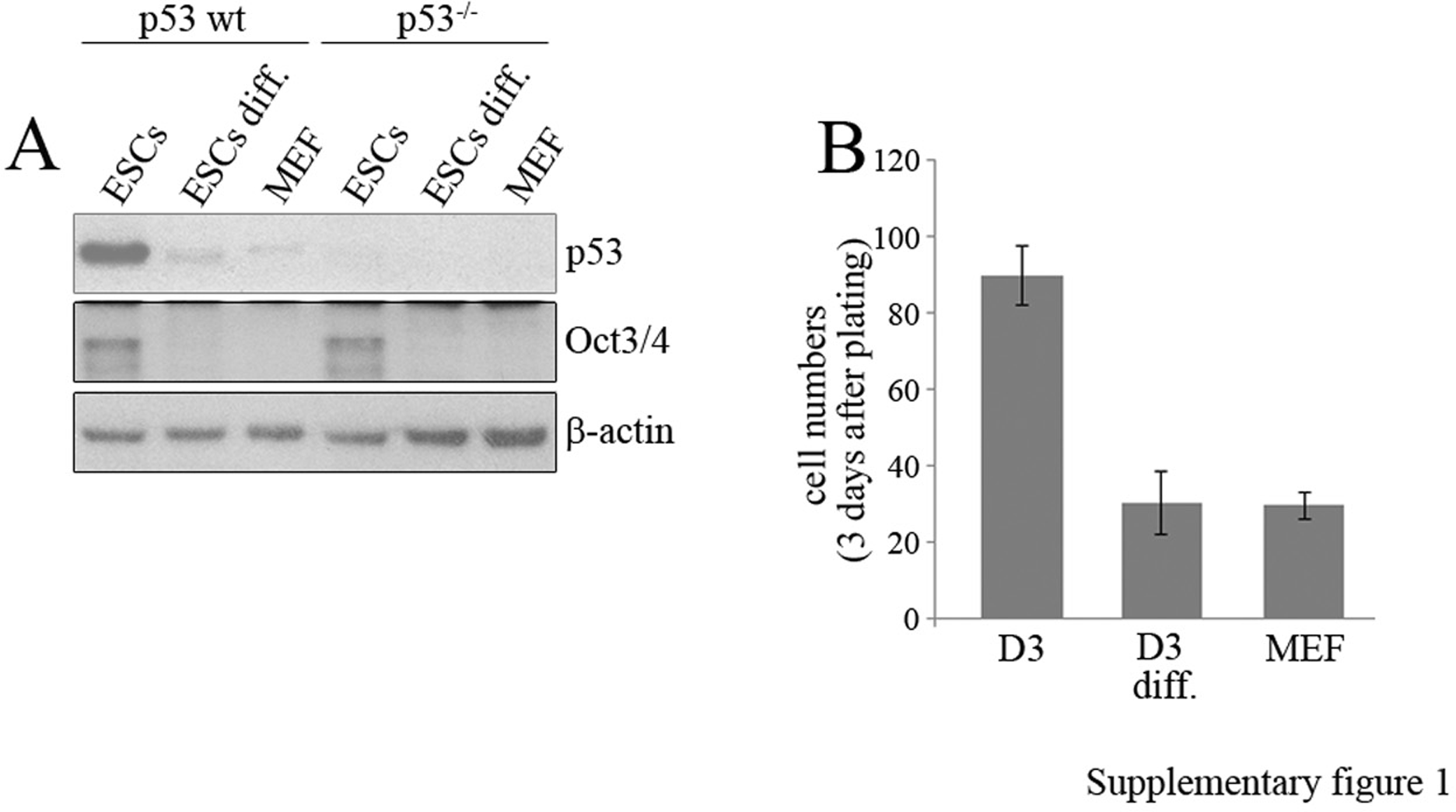

Supplement: Supplementary Figure 1 [file cddis201533x1.tif]

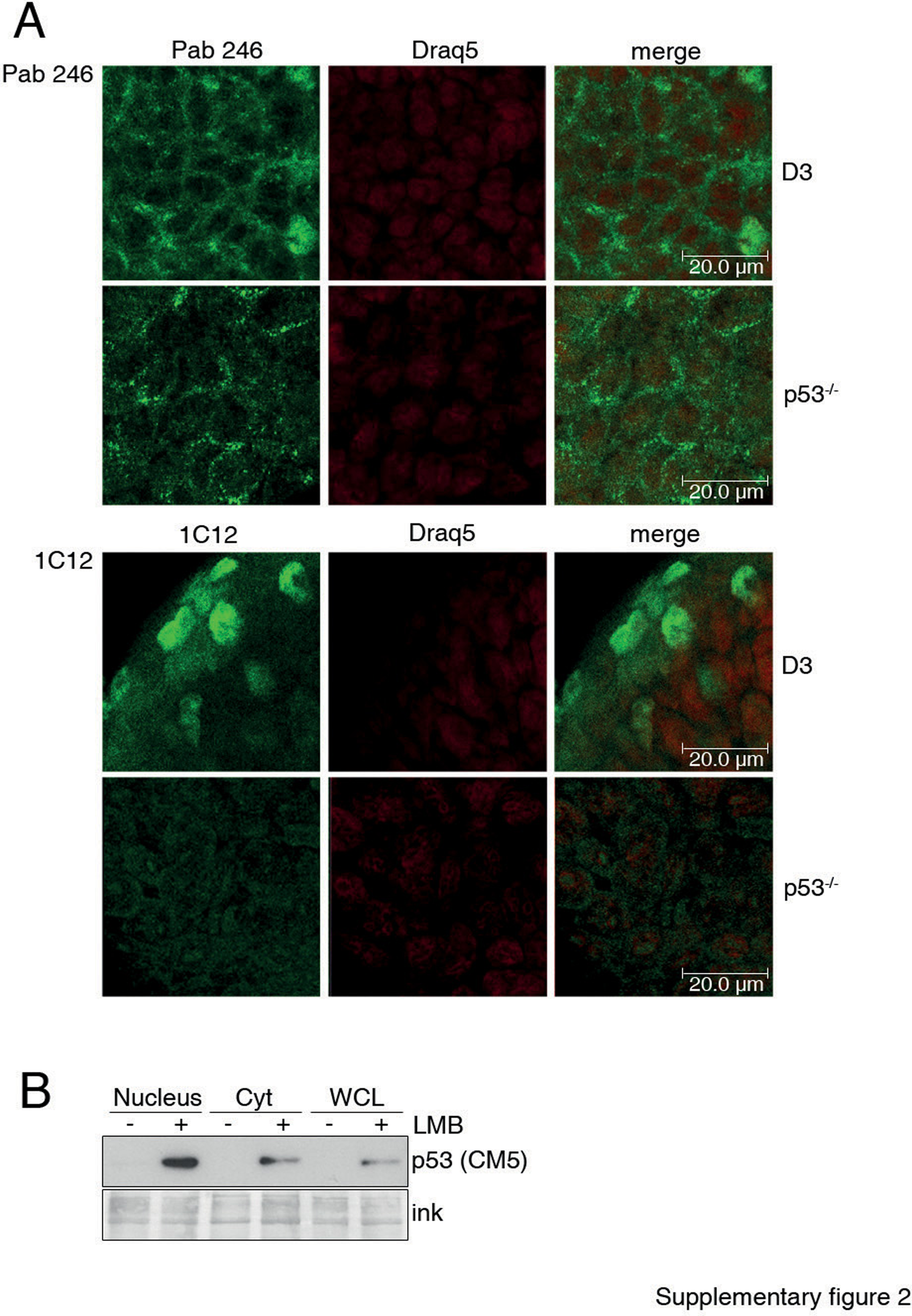

Supplement: Supplementary Figure 2 [file cddis201533x2.tif]

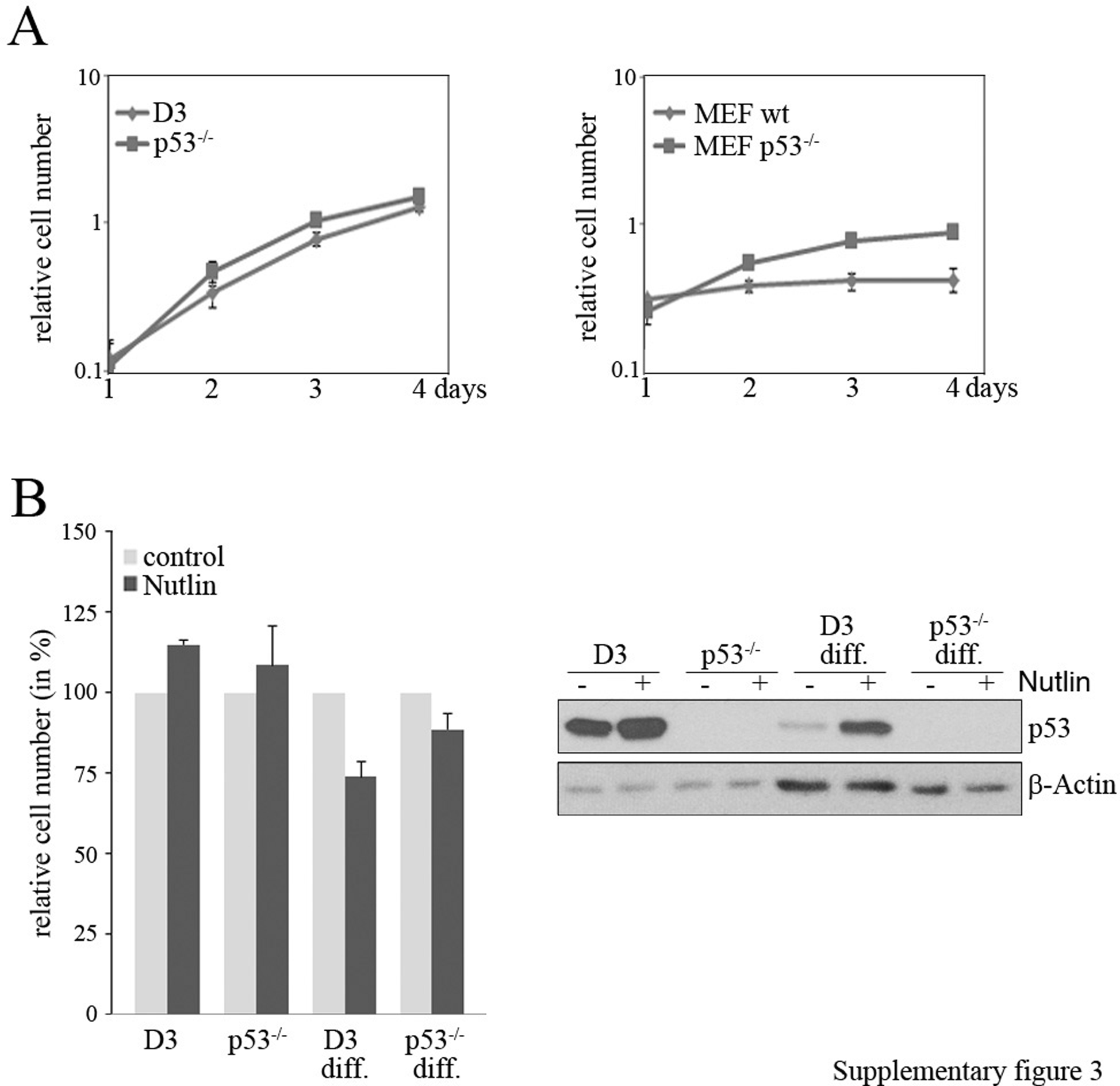

Supplement: Supplementary Figure 3 [file cddis201533x3.tif]

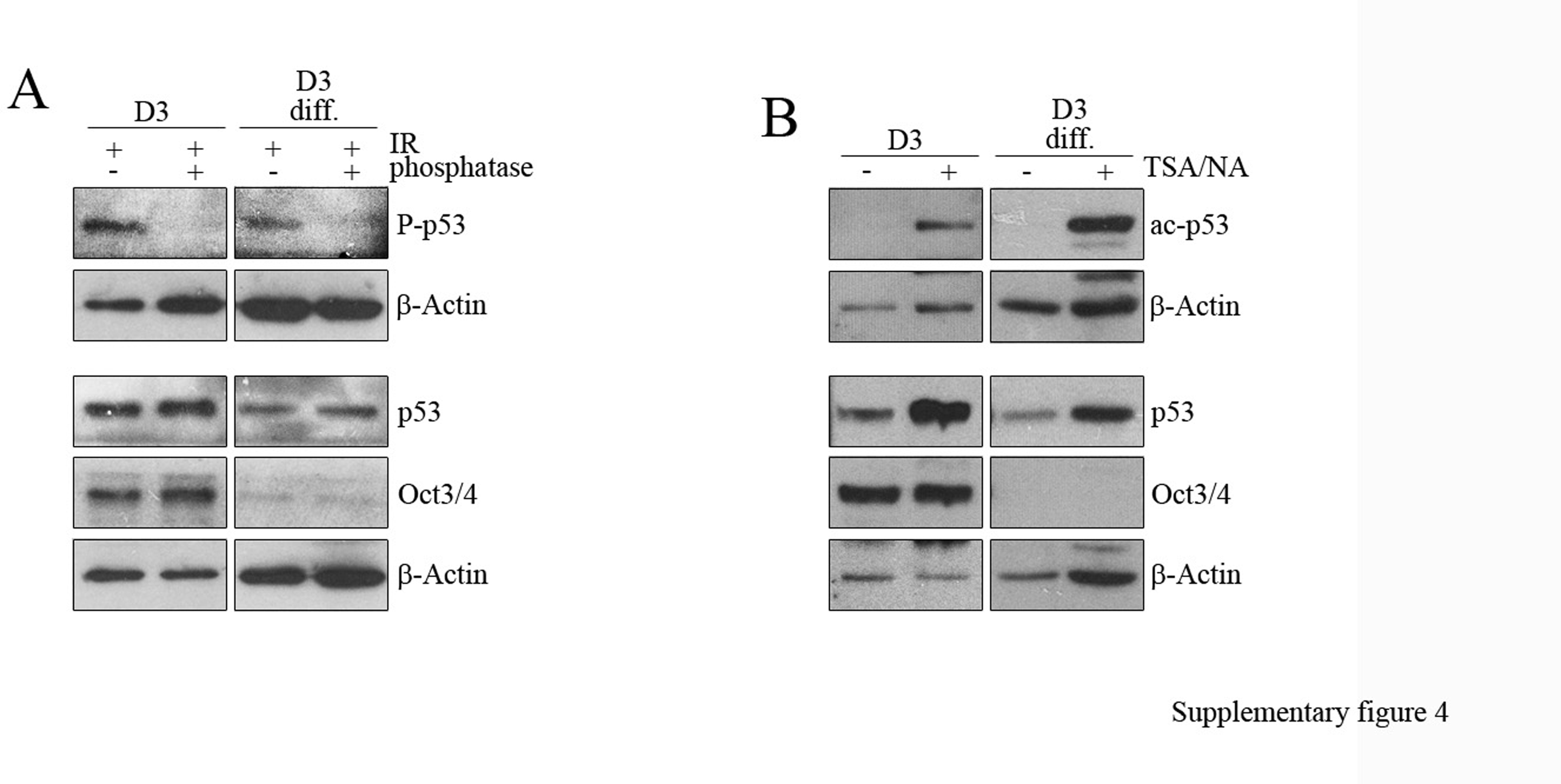

Supplement: Supplementary Figure 4 [file cddis201533x4.tif]

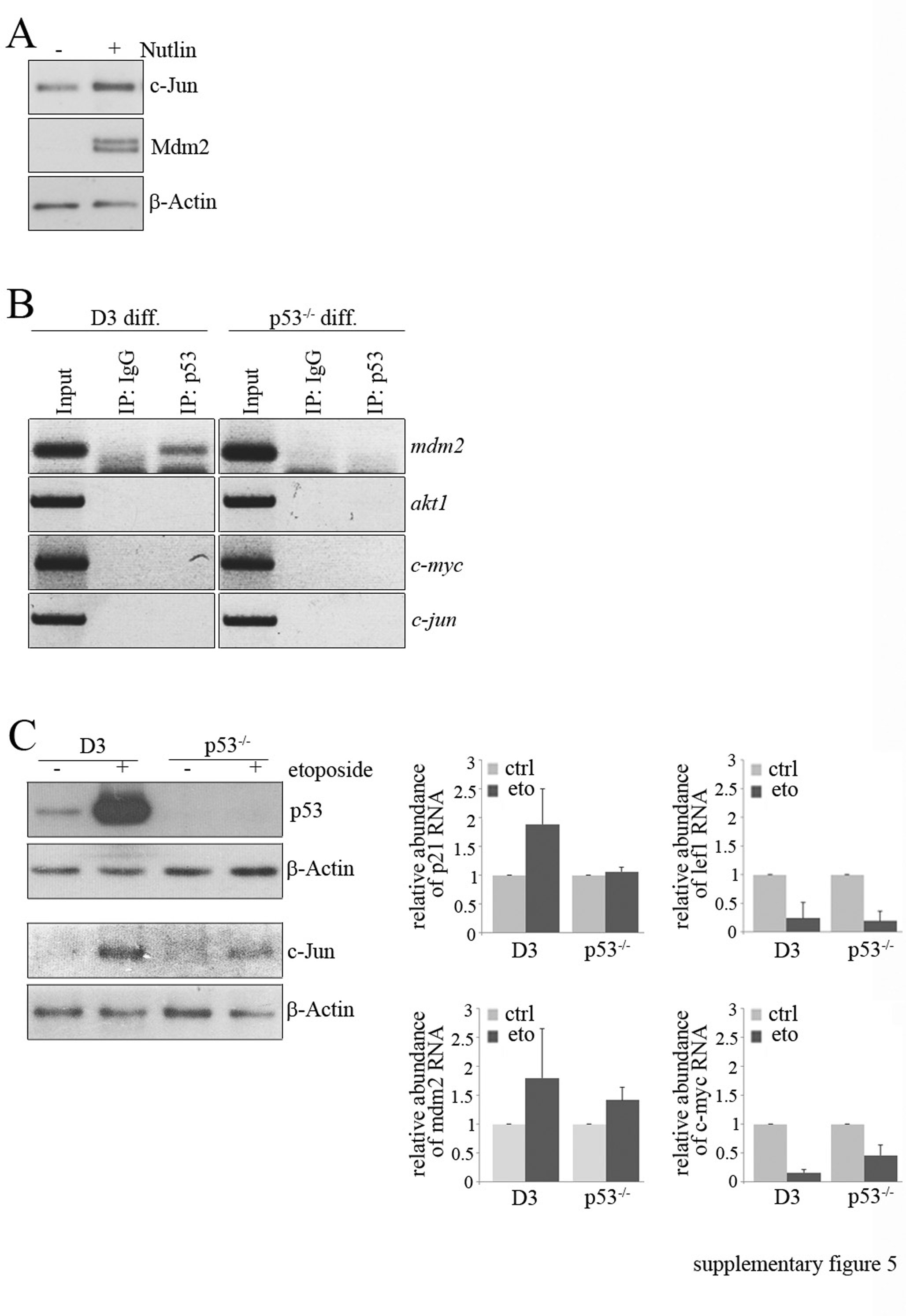

Supplement: Supplementary Figure 5 [file cddis201533x5.tif]

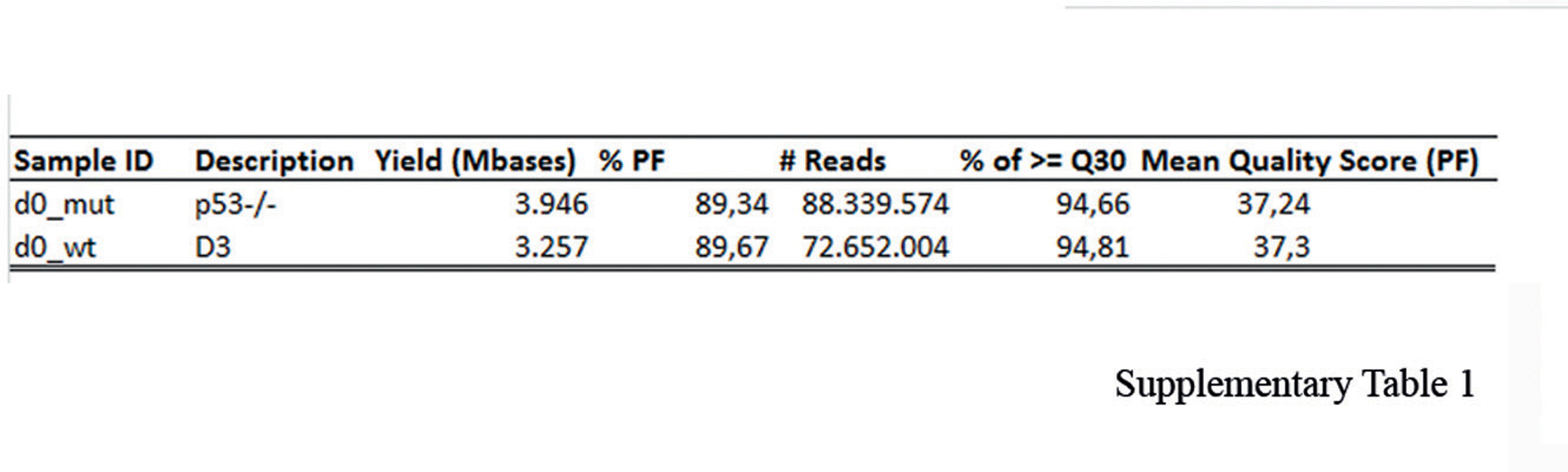

Supplement: Supplementary Table 1 [file cddis201533x6.tif]
