# Supplementary material for: p53 is active in murine stem cells and alters the transcriptome in a manner that is reminiscent of mutant p53
Source: Cell Death Dis. 2015 Feb 26;6(2):e1662–. doi: 10.1038/cddis.2015.33 (PMC4669809; doi:10.1038/cddis.2015.33)
Supplement: Supplementary Table 2 [file cddis201533x7.doc]

| **Gene** | **Primer sequence** | **Cycle conditions** | **Number**  **of cycles** |
| --- | --- | --- | --- |
| Mdm2 | 5’-cag gag gtg aca ggt gcc-3’  5’-cag gac tta gct cct ccg ac-3’ | 95°C - 2 min;  95°C - 20 sec; 55°C - 20 sec; 72°C - 30 sec;  72°C – 5 min; | 53 |
| Akt-1 | 5’-cca agc ctc acc cat ctg a-3’  5’-gcg tgg gaa gtg aat cag ttt-3’ | 95°C - 2 min;  95°C - 20 sec; 55°C - 20 sec; 72°C - 30 sec;  72°C – 5 min; | 60 |
| c-Jun | 5’-tcc gac aga ctc cgc aag-3’  5’-tga gtc ctt atc cag cctg ag-3’ | 95°C - 2 min;  95°C - 20 sec; 55°C - 20 sec; 72°C - 30 sec;  72°C – 5 min; | 62 |
| c-Myc | 5’-gta agc aca gat ctg gtg g-3’  5’-tgg taa gtc aga agc tacg ga-3’ | 95°C - 2 min;  95°C - 20 sec; 55°C - 20 sec; 72°C - 30 sec;  72°C – 5 min; | 75 |

Supplementary Table 2
