# Supplementary material for: p53 is active in murine stem cells and alters the transcriptome in a manner that is reminiscent of mutant p53
Source: Cell Death Dis. 2015 Feb 26;6(2):e1662–. doi: 10.1038/cddis.2015.33 (PMC4669809; doi:10.1038/cddis.2015.33)
Supplement: Supplementary Table 3 [file cddis201533x8.doc]

**:**

| **Gene** | **Primer sequence** |
| --- | --- |
| *mdm2* | for: tgg agt ccc gag ttt ctc tg  rev: agc cac taa att tct gta gat cat tg |
| *mdmX* | for: cca tct gac gac atg ttt cc  rev: tta caa gca gga cac gaa gc |
| *p53* | for: cct cat cct cct cct tcc cag cag  rev: aac aga tcg tcc atg cag tga ggt g |
| *p21* | for: cct gac aga ttt cta tca ctc ca  rev: cag gca gcg tat atc agg ag |
| *puma* | for: ggc gga tgg cgg acg acc t  rev: ggc ggg tcc cat gaa gag att gta c |
| *lef-1* | for: ccc aca cgg aca gtg acc ta  rev: tgg gct cct gct cct ttc t |
| *c-jun* | for: cga gta ctg aag cca agg gta cac  rev:tga gat cga atg tta ggt cca tgc a |
| *c-myc* | for: gct gta gta att cca gcg aga gac  rev: ctc tgc aca cac ggc tct tc |
| *akt-1* | for: tgc att gcc gag tcc aga a  rev: cag cgc atc cga gaa aca |
| *igf-2* | for: cgt ggc atc gtg gaa gag t  rev: aca cgt ccc tct cgg act tg |
| *ribpo* | for: gaa ggc tgt ggt gct gat gg  rev: ccg gat atg agg cag cag |

Supplementary Table 3
