# Supplementary material for: p53 is active in murine stem cells and alters the transcriptome in a manner that is reminiscent of mutant p53
Source: Cell Death Dis. 2015 Feb 26;6(2):e1662–. doi: 10.1038/cddis.2015.33 (PMC4669809; doi:10.1038/cddis.2015.33)
Supplement: Supplementary Figures and Table Legends [file cddis201533x9.doc]

**Figure S1:** **(A)** D3 cells, D3 cells that had been differentiated with retinoic acid (D3 diff.), mouse embryonic fibroblasts (MEF) and their p53-deficient counterparts were lysed and abundance of p53 was monitored by Western blotting. The amount of Oct3/4 was monitored to control for stemness and the amount of β-actin for loading control. **(B)** D3 cells and mouse embryonic fibroblasts (MEF) were plated at a density of 5 x 10^4^ cells/well in a 6-well plate. Retinoic acid was added at the time of plating. Cells were counted three days after plating. The graph shows mean values and standard deviations of three independent experiments.

**Figure S2: The majority of p53 is localised in the nucleus in murine embryonic stem cells. (A)** D3 embryonic stem cells and their p53-deficient derivative (p53^-/-^) were grown on feeder cells on coverslips. Cells were fixed, stained with the indicated antibodies (shown in green) and counterstained with the nuclear marker Draq5 (shown in red). Images were analysed on a Zeiss SP5 microscope **(B)** Cells were serum starved for 24 hours prior to treatment with 2 µM leptomycin B (LMB) for 16 hours. An aliquot of the cells was lysed and used to monitor abundance of p53 in the whole cell lysate (WCL). The remaining cells were fractionated into cytoplasmic and nuclear lysate. 40 µg of the different fractions were loaded onto an SDS-PAGE gel and blotted. The membrane was hybridised with the anti-p53 antibody CM5. Staining with ink was performed to monitor equal loading of the gel.

**Figure S3:** **The anti-proliferative activity of p53 is compromised in stem cells.** **(A)** D3 cells, their p53-deficient derivative (p53^-/-^), mouse embryonic fibroblasts (MEF) and their p53-deficient counterpart (MEF p53^-/-^) were plated at a density of 5 x 10^4^ cells/well in a 6-well plate. At one, two, three and four days after plating, MTT-assays were performed. The graph shows mean values and standard deviations of three independent experiments. **(B)** D3 cells, D3 cells that had been differentiated with retinoic acid (D3 diff.), and their p53-deficient counterparts (p53^-/-^ and p53^-/-^ diff) were plated in quadruplicates and treated with 5 µM nutlin or with DMSO for control (with a daily change of the culture medium). 3 days after plating, relative cell proliferation was assessed from triplicates by MTT assay. The graph shows mean values and error bars of two independent experiments. Relative cell numbers of mock treated cells were set to 100 %. The remaining part of the quadruple was lysed 32 hours after nutlin treatment and abundance of p53 was assessed by Western Blotting. Hybridisation with β-Actin was performed for loading control.

**Figure S4: Inhibition of HDACs leads to an increase in p53 abundance. (A)** D3 cells (ESC) and D3 cells that had been differentiated with retinoic acid for 7 days (D3 Diff.) were irradiated with 10 Gray and harvested after 30 minutes. Cells were suspended in phosphatase buffer and lysed by sonication. Lambda phosphatase was added at a concentration of 200 units per 100 µg of protein (or cells were left without phosphatase for control) and cells were incubated for 30 minutes at 30°C. 50 µg of protein were separated by SDS-PAGE gels and blotted. Abundance of phosphorylated p53, total p53, Oct3/4 and β-Actin, for internal control, was determined by Western Blotting. **(B)** D3 cells (ESC) and D3 cells that had been differentiated with retinoic acid for 7 days (D3 Diff.) were treated with 1 µM trichostatin A (TSA) and 5 mM nicotinamide (NA) for 6 hours. Cells were lysed, separated on a SDS-PAGE gel and blotted. Abundance of acetylated p53, total p53, Oct 3/4 and of β-Actin, for internal control, was monitored by Western Blotting.

**Figure S5: p53 does not bind to *c-myc*, *c-jun* or *akt-1* DNA in differentiated cells.** **(A)** D3 cells were treated with 20 µM Nutlin for 4 hours. Cells were lysed and abundance of c-Jun and Mdm2 was monitored by Western Blotting. β-Actin was used for loading control **(B)** Differentiated D3 cells (D3 diff.) and p53-deficient differentiated cells (p53^-/-^ diff.), were lysed. p53 was precipitated (IP: p53) and associated *c-myc*, *c-jun*, *akt-1* and *mdm2* DNA was monitored by PCR. Precipitation with IgG and total cell lysate (Input) were used for positive and negative control. **(C)** D3 cells and their p53-negative counterpart (p53-/-) were treated with 50 µM etoposide or with ethanol for control. 4.5 hours after treatment, cells were harvested. An aliquot of the cells was used for Western blotting to monitor abundance of p53 and c-Jun. From the remaining cells, RNA was prepared and abundance of p21, mdm2, lef1 and c-myc was determined by qRT-PCR. Abundance of specific cDNAs was normalised by the abundance of the housekeeping gene RibPO. The graph shows mean values and error bars of two to three independent experiments. Relative abundance of the specific RNA in mock-treated cells was set to 1

**Table S1: Quality of the RNAseq generated.** RNA was prepared from ESCs possessing wild-type p53 (D3) and ESCs where p53 was genetically deleted (p53^-/-^). The RNA was transcribed into cDNA and subjected to RNA sequencing. (PF: clusters passing illumine chastity filter.)

**Table S2: Primers and cycle conditions for CHIP**

**Table S2: Primers for qRT-PCR**
